# Teledermatology: an evidence map of systematic reviews

**DOI:** 10.1186/s13643-024-02655-5

**Published:** 2024-10-12

**Authors:** Aloysius Chow, Helen Elizabeth Smith, Lorainne Tudor Car, Jing Wen Kong, Kay Wee Choo, Angeline Ai Ling Aw, Marie Ann Mae En Wong, Christian Apfelbacher

**Affiliations:** 1 Family Medicine and Primary Care, Lee Kong Chian School of Medicine, Nanyang Technological University Singapore, Singapore, Singapore; 2 Institute of Social Medicine and Health Systems Research, Otto von Guericke University Magdeburg, Leipziger Str. 44, Bldg 2, office 120, Magdeburg, 39120 Germany; 3 National Healthcare Group Polyclinics, Singapore, Singapore; 4 A Life Clinic Pte Ltd, Singapore, Singapore; 5 School of Medicine, Keele University, Staffordshire, UK

**Keywords:** Teledermatology, Telemedicine, Dermatology, Evidence map, Systematic review

## Abstract

**Background:**

Although the number of teledermatology studies is increasing, not all variables have been researched in equal depth, so there remains a lack of robust evidence for some teledermatology initiatives. This review describes the landscape of teledermatology research and identifies knowledge gaps and research needs. This evidence map can be used to inform clinicians about the current knowledge about teledermatology and guide researchers for future studies.

**Methods:**

Our evidence map was conducted according to the Campbell Collaboration checklist for evidence and gap maps. Eight databases were searched (CINAHL, Embase, PubMed, Scopus, Web of Science, Cochrane Library, JBI Database of Systematic Reviews and Implementation Reports, and OpenGray), and only included systematic reviews of teledermatology involving humans published in English; while excluding non-systematic reviews (i.e., abstracts, conference proceedings, editorials, commentaries, or letters). From 909 records, 14 systematic reviews published between 2004 and 2022 were included. Our analysis focused on the systematic reviews’ characteristics, dermatological conditions studied, rate of overlap and quality assessment of primary studies reviewed, and main findings reported.

**Results:**

Teledermatology was reportedly comparable with clinic dermatology and generally accepted by patients as a mode of care delivery for dermatological conditions. However, there are concerns about privacy, communication, completeness of information transmitted, familiarity with the technology, and technical problems. Healthcare professionals were generally satisfied with teledermatology but found telemedicine consultations longer than face-to-face consultations, and less confident in asynchronous teledermatology than conventional consultations. Teledermatology was reportedly more cost-effective than clinic dermatology; especially considering the distance traveled by patients, referral volume to teledermatology, and clinic dermatology costs. Although patients and providers are satisfied with teledermatology, face-to-face dermatology has higher diagnostic and management accuracy. Teledermatology was also used for training medical professionals. Regarding the validity and reliability of teledermatology outcome measures, no significant discussions were found.

**Conclusions:**

COVID-19 spotlighted telemedicine in clinical care, and we must ensure telemedicine continually improves with robust research. Further research is necessary for establishing a standardized outcome set, enhancing accuracy, concordance, cost-effectiveness, and safety, comparing teledermatology with non-dermatologist care, examining its effectiveness in non-Western low and middle-income countries, and incorporating patient involvement for improved study design.

**Systematic review registration:**

https://www.researchregistry.com/ (Unique Identifying Number: reviewregistry878).

**Supplementary Information:**

The online version contains supplementary material available at 10.1186/s13643-024-02655-5.

## Introduction

Teledermatology has been introduced in the hope of increasing access to care and improving health outcomes for patients while reducing healthcare costs to both patients and providers [1]. With the proliferation of the internet and advancements in technology, telemedicine has been implemented in a wide number of clinical specialties and institutions. The main modes of teledermatology consultations are the transmission of digital photographs for review (referred to as asynchronous or store and forward (SF)) or real-time (referred to as synchronous, live-interactive (LI), or face-to-face virtual communication): sometimes with methods used in combination [1]. Telemedicine has been used for a wide range of dermatological conditions (e.g., acne, melanoma, psoriasis) and for different populations in the community (e.g., children, older people, military veterans) [2–5].

A key advantage of using teledermatology is to remove physical or geographical barriers to dermatologic care for patients who would otherwise have difficulty accessing such care. Another advantage of teledermatology, as perceived by teledermatologists, is the ability to help patients who would find it costly to have a face-to-face consultation [6]. Other reported benefits include shorter waiting times for patients to receive a diagnosis and management [7], whilst achieving diagnostic and treatment concordance with face-to-face consultations [8]. Economic evaluations have demonstrated that a teledermatology consultation can be more cost-effective than a face-to-face consultation [9, 10].

There is an increasing amount of literature evaluating heterogeneous interventions for teledermatology, with services delivered to various participants in diverse settings in different ways. This growth in research is evident when searching “teledermatology” on the PubMed database with 70 records between 1995 (i.e., the start of PubMed was in 1996) and 2000, 240 records from 2001 to 2010, and 700 records between 2011 and 2020, and 697 records in the three and half years between 2021 and June 2024. At the same time, there is a lack of robust evidence for some teledermatology applications as not all conditions, settings, approaches, and patient groups have been researched in equal measure. With the growing number of systematic reviews of teledermatology, it is beneficial to map the available evidence, identifying gaps in the literature and research needs. Our evidence map aimed to describe the landscape of teledermatology research by mapping the existing evidence in systematic reviews.

## Methods

The review was registered at https://www.researchregistry.com/(Unique Identifying Number: reviewregistry878). The Campbell Evidence and Gap Map conduct standards [11] were used for methodological guidance.

### Search strategy

The search included articles published between 01st January 2004 and 31st January 2023, from five databases (CINAHL, Embase, PubMed, Scopus, and Web of Science), two systematic review repositories (Cochrane Library and JBI Database of Systematic Reviews and Implementation Reports), and the grey literature database OpenGray. The search strategies used are shown in Appendix 1. Searches were supplemented by screening the reference lists of review articles.

### Inclusion and exclusion criteria

Any systematic review of teledermatology involving humans, with or without meta-analysis, and published in English was considered eligible for inclusion. Reviews were excluded if they were non-systematic reviews (e.g., narrative reviews) or if they were abstracts, conference and meeting proceedings, editorials, commentaries, or letters. Included SRs were classified using a typology of systematic reviews [12]. Those that were specifically designed to explore the breadth or depth of literature, map and summarize evidence, or identify knowledge gaps were classified as scoping reviews [13].

### Screening and selection of systematic reviews

Two reviewers independently screened the titles and abstracts of citations to remove duplicates and citations that did not meet the inclusion criteria and assessed the eligibility of the full-text articles. Disagreements between reviewers were resolved by discussion with a senior author.

### Data extraction

Two reviewers independently extracted the following data into a spreadsheet: (1) reference of systematic review; (2) systematic review publication includes statement about prior registration or publication of a protocol (i.e., yes or no); (3) focus of systematic review (i.e., interventions, diagnostic test accuracy, qualitative studies, observational studies, outcomes, outcome measures); (4) conflict of interest declared as stated in the publication (i.e., conflict, no conflict, or no comment); (5) funding statement (i.e., yes or no); (6) information source (e.g., electronic bibliographies, trials registries); (7) aim and research question of the systematic review; (8) dermatological conditions included in the systematic review; (9) number of included primary studies; (10) study designs of included primary studies; (11) details of research participants in included primary studies (gender, number of adults (i.e. ≥ 18 years) and children participants, range, mean, median, standard deviation); (12) quality assessment of studies by systematic review (instrument used for quality assessment and the findings from the quality assessment); and (13) main findings of the systematic review. As before, disagreements were discussed with a senior author. The data are presented as frequencies where appropriate.

### Overlap of primary studies

The overlap of primary studies included in two or more systematic reviews was analyzed using the corrected covered area (CCA) [14]. The CCA measures the amount of overlap by dividing the frequency of repeated occurrences of the primary study in other systematic reviews by the product of the total number of primary studies and the total number of systematic reviews, reduced by the number of primary studies. The corrected covered area is an indicator of the amount of overlap (i.e., <  = 5% indicating a slight overlap, 6% to 10% indicating a moderate overlap, 11% to 15% indicating a high overlap, and more than 15% indicating a very high overlap). To further analyze the CCA, reviews were grouped into pairs. We used the GROOVE (Graphical Representation of Overlap for OVErviews) tool for this calculation [15].

## Results

**Fig. 1:**
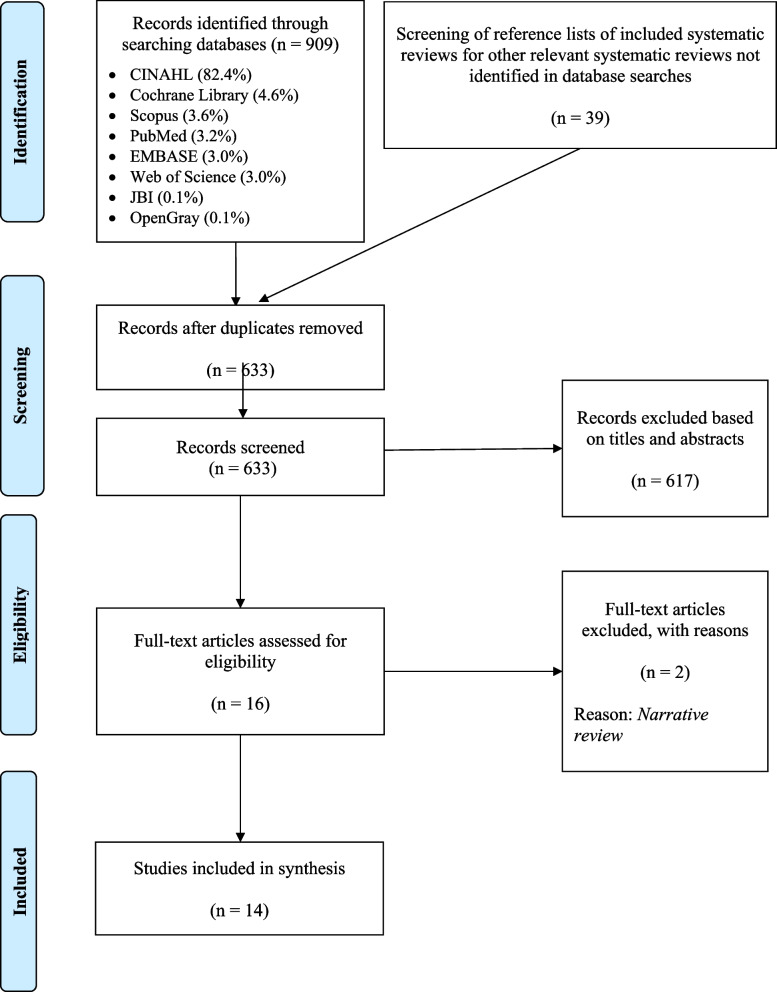
PRISMA 2009 flow diagram

We included fourteen systematic reviews published between 2004 and 2023 were finally included in this evidence map [9, 16–28]. Figure 1 shows the Preferred Reporting Items for Systematic Reviews and Meta-Analyses (PRISMA) flow diagram [29].

### Characteristics of included systematic reviews

Table 1 shows the types of SRs included. Two were costs/economic evaluation reviews, two diagnostic test accuracy reviews, one experiential review, one a combination of experiential and psychometric, one combined diagnostic test accuracy with a costs/economic evaluation, and seven scoping reviews.

**Table 1:** Characteristics of included systematic reviews

**Reference of systematic review**	**Year of Publication**	**Country**	**Type of systematic Review**	**Aim(s) of the systematic review**	**Dermatological conditions included in the systematic review**	**Total Number of included primary studies**
Fuertes-Guiro, F., & Girabent-Farrés, M. (2017). Opportunity cost of the dermatologist’s consulting time in the economic evaluation of teledermatology. *Journal of Telemedicine and Telecare*, *23*(*7*), 657-664.	2017	Spain	• Costs/Economic Evaluation	To evaluate the opportunity cost through an economic evaluation of teledermatology consultation and conventional dermatology consultation.	Not mentioned in systematic review	8
van der Heijden, J. P., Spuls, P. I., Voorbraak, F. P., de Keizer, N. F., Witkamp, L., & Bos, J. D. (2010). Tertiary teledermatology: a systematic review. *Telemedicine and e-health*, *16*(*1*), 56-62.	2010	The Netherlands	• Scoping review	To provide an overview of tertiary teledermatology studies focusing on what tertiary teledermatology is used for and to compare tertiary teledermatology with secondary teledermatology.	Not mentioned in systematic review	11
Snoswell, C., Finnane, A., Janda, M., Soyer, H. P., & Whitty, J. A. (2016). Cost-effectiveness of store-and-forward teledermatology: a systematic review.* JAMA Dermatology*, *152*(*6*), 702-708.	2016	Australia	• Costs/Economic Evaluation	To evaluate and compare the cost effectiveness of store-and-forward teledermatology with conventional face-to-face care.	Psoriasis, suspected cancer, ambulatory skin conditions, nonmelanoma skin cancer or fast-growth vascular tumours, and some were not specified.	11
Demiris, G., Speedie, S. M., & Hicks, L. L. (2004). Assessment of patients' acceptance of and satisfaction with teledermatology. *Journal of medical systems*, *28*(6), 575-579.	2004	USA	• Experiential • Psychometric	To review and analyse published literature and measurements of patients’ satisfaction with teledermatology to propose and develop a framework for a reliable and valid satisfaction instrument	Not mentioned in systematic review	14
Chuchu, N., Dinnes, J., Takwoingi, Y., Matin, R. N., Bayliss, S. E., Davenport, C., ... & Walter, F. M. (2018). Teledermatology for diagnosing skin cancer in adults. *Cochrane Database of Systematic Reviews*, *12*.	2018	United Kingdom	• Diagnostic Test Accuracy	To assess whether teledermatology is accurate enough to identify which people with skin lesions require referrals to a dermatologist to evaluate whether the lesion is malignant.	Skin cancer	22
Wallace, D. L., Hussain, A., Khan, N., & Wilson, Y. T. (2012). A systematic review of the evidence for telemedicine in burn care: with a UK perspective. *Burns*, *38*(*4*), 465-480.	2012	United Kingdom	• Scoping review	To assess the evidence for the use of telemedicine in acute burn care and outpatient-based management.	Skin burns	24
Clark, A. K., Bosanac, S., Ho, B., & Sivamani, R. K. (2018). Systematic review of mobile phone-based teledermatology. *Archives of dermatological research*, *310*(*9*), 675-689.	2018	USA	• Diagnostic Test Accuracy	To provide an overview of the mobile phone-based teledermatology, to compare the accuracy and concordance of diagnosis and clinical management of skin conditions between mobile teledermatology and face-to-face dermatology, and to assess how data was managed in teledermatology studies.	Not mentioned in systematic review	26
Mounessa, J. S., Chapman, S., Braunberger, T., Qin, R., Lipoff, J. B., Dellavalle, R. P., & Dunnick, C. A. (2018). A systematic review of satisfaction with teledermatology. *Journal of Telemedicine and Telecare*, *24*(*4*), 263-270.	2018	USA	• Experiential	To review assessments of patient and provider satisfaction with store-and-forward and live-interactive teledermatology.	Not mentioned in systematic review	40
Warshaw, E. M., Hillman, Y. J., Greer, N. L., Hagel, E. M., MacDonald, R., Rutks, I. R., & Wilt, T. J. (2011). Teledermatology for diagnosis and management of skin conditions: a systematic review. *Journal of the American Academy of Dermatology*, *64*(*4*), 759-772.	2011	USA	• Costs/Economic Evaluation • Diagnostic Test Accuracy	To compare the diagnostic accuracy and clinical management of skin conditions between teledermatology and clinic dermatology, to compare the clinical outcomes between teledermatology and clinic dermatology, and to compare the cost between teledermatology and clinic dermatology.	R*ashes (e.g. papulosquamous, eczematous) and circumscribed lesions (isolated skin growths), pigmented, nonpigmented, and circumscribed lesions.*	78
Eminović, N., De Keizer, N. F., Bindels, P. J. E., & Hasman, A. (2007). Maturity of teledermatology evaluation research: a systematic literature review. *British Journal of Dermatology*, *156*(*3*), 412-419.	2007	The Netherlands	• Scoping review	To describe the maturity status of teledermatology evaluation research and to explore the outcome measures used in the various evaluation phases.	Not mentioned in systematic review	99
Trettel, A., Eissing, L., & Augustin, M. (2018). Telemedicine in dermatology: findings and experiences worldwide–a systematic literature review. *Journal of the European Academy of Dermatology and Venereology*, *32*(*2*), 215-224.	2018	Germany	• Scoping review	To identify the use and current state of teledermatology across the world with regard to geographical distribution of published studies, treated indications, research questions, and its reliability in diagnosis and therapy compared to classic face-to-face consultations.	Skin cancer, wounds, psoriasis, atopic dermatitis, acne, leprosy, rash, tinea, and some were not specified.	204
Elsner, P. (2020). Teledermatology in the times of COVID‐19–a systematic review. *JDDG: Journal der Deutschen Dermatologischen Gesellschaft*, *18*(*8*), 841-845.	2020	Germany	• Scoping review	To summarise teledermatological procedures used by dermatologists and the experiences of using teledermatological procedures in dermatological practices and clinics during the COVID-19 pandemic.	Acne, chronic inflammatory dermatoses, dermatological consultations for suspected COIVD-19, and dermatologic complications in oncologic patients	7
Loh, C. H., Chong Tam, S. Y., & Oh, C. C. (2021). Teledermatology in the COVID-19 pandemic: A systematic review. *JAAD Int*, 5, 54-64.	2021	Singapore	• Scoping review	To analyse and report the worldwide utilisation of teledermatology for patient care during the COVID-19 pandemic.	Not mentioned in systematic review	27
Miller, J., & Jones, E. (2022). Shaping the future of teledermatology: a literature review of patient and provider satisfaction with synchronous teledermatology during the COVID-19 pandemic. *Clin Exp Dermatol*.;*47*(*11*):1903-9.	2022	USA	• Scoping review	To identify the patient and provider satisfaction levels of synchronous teledermatology used during the COVID-19 pandemic	Not mentioned in systematic review	15

Characteristics of the included systematic reviews are shown in Table 2. The reviews were undertaken in Europe, the USA, Australia, and Singapore.

**Table 2:** Inclusion and exclusion criteria used in systematic reviews

**Reference of systematic review**	**Date range of publication searched**	**Electronic database(s) used**	**Inclusion criteria**	**Exclusion criteria**
Demiris, G., Speedie, S. M., & Hicks, L. L. (2004). Assessment of patients' acceptance of and satisfaction with teledermatology. *Journal of medical systems*, *28*(6), 575-579.	1966 to 2003	1) Embase 2) Medline 3) Science Citation Index 4) Telemedicine Information Exchange	1) Studies published in English 2) Studies that used quantitative and/or qualitative methods to investigate patient satisfaction with teledermatology applications in a prospective or retrospective manner	1) Reviews and concept papers
Eminović, N., De Keizer, N. F., Bindels, P. J. E., & Hasman, A. (2007). Maturity of teledermatology evaluation research: a systematic literature review. *British Journal of Dermatology*, *156*(*3*), 412-419.	1966 to 2006	1) Medline	1) Studies published in English 2) Original full papers reporting on the evaluation of a specific teledermatology service 3) Papers on a telemedicine application for several specialties (*i.e. only if the results on dermatology were separately reported*)	1) Literature reviews, comments, abstracts, letters, and editorials 2) Papers not about dermatology but about another specialty (*e.g. radiology, pathology*) 3) Papers where the evaluation of a specific teledermatology service was not the primary aim 4) Papers from conference proceedings were excluded if a full journal paper on the same study was obtained in the selection procedure
van der Heijden, J. P., Spuls, P. I., Voorbraak, F. P., de Keizer, N. F., Witkamp, L., & Bos, J. D. (2010). Tertiary teledermatology: a systematic review. *Telemedicine and e-health*, *16*(*1*), 56-62.	No limit reported	1) Cochrane Library 2) Medline3) Scopus	1) All articles on tertiary teledermatology, including original research, comments, letters, and editorials. 2) No language restrictions 3) During the title scan, references were included if one of the words ‘‘teledermatology,’’ ‘‘dermatol*,’’ or ‘‘skin*’’ was found in the title. References with the word ‘‘telemedicine’’ in the title were only included if no specialty (other than dermatology) was mentioned in the title.4) References without an abstract in the database were subject to a second title scan, where references were only included if the title included the word ‘‘teledermatology’’ 5) During the full text screening, references were included if the main subject of the article was the use of teledermatology between dermatologists, or a dermatology resident and a specialized dermatologist.	1) Conference proceedings and errata 2) During the full text screening, references were excluded if the referrer was a primary care physician or specialist other than a dermatologist, the article was excluded
Warshaw, E. M., Hillman, Y. J., Greer, N. L., Hagel, E. M., MacDonald, R., Rutks, I. R., & Wilt, T. J. (2011). Teledermatology for diagnosis and management of skin conditions: a systematic review. *Journal of the American Academy of Dermatology*, *64*(*4*), 759-772.	1990 to 2009	1) Medline 2) PubMed	1) Clinical trials, systematic reviews, cost studies, and implementation papers involving human participants 2) Controlled trial studies3) Store and forward or live interactive teledermatology studies 4) Clinical trials of teledermatology with a clinic dermatology control group (in-person examination) if they provided information related to diagnostic and management accuracy or concordance as defined by the authors 5) Teledermatology studies without control groups that compare clinical outcomes (i.e. clinical course, satisfaction, quality of life, visits avoided) with clinic dermatology 6) Teledermatology studies without control groups that compare the cost with clinic dermatology	1) Teledermatology involving mobile telephones. 2) Nonteledermatology settings (e.g., imaging analyses, telemedicine studies other than teledermatology, videomicroscopy studies, basic science, imaging techniques) 3) Dermatopathology studies 4) Reviews, teledermatology program descriptions, and historical summaries of teledermatology (unless relevant to questions 3 or 4) 5) Studies of computer-aided diagnoses only (e.g., computerized pattern recognition for pigmented lesions) 6) Survey studies addressing outcomes other than those defined research questions 7) Teledermatology as an educational tool for primary care physicians or residents 8) Technology assessment only 9) Remote monitoring of known diagnoses (e.g., leg ulcers, postoperative wounds) 10) Teledermatology involving patient-generated photographs, history, or both (without a referring provider) 11) Non-English language 12) Case series with no control group (questions 1 and 2 only) 13) Commentaries, editorials, or meeting abstracts 14) Studies involving only one or two diagnoses (of, leprosy, acne, warts); studies of one category of skin conditions (e.g., pigmented lesions that could have multiple diagnoses) were included 15) Duplicate publications; if both preliminary and final reports were published, final datawere used
Wallace, D. L., Hussain, A., Khan, N., & Wilson, Y. T. (2012). A systematic review of the evidence for telemedicine in burn care: with a UK perspective. *Burns*, *38*(*4*), 465-480.	1966 to 2010	1) Arts and Humanities Citation Index 2) CINAHL 3) Cochrane Controlled Trials Register 4) EMBASE 5) Medline 6) Science Citation Index 7) Social Sciences Citation Index 8) Telemedicine Information Exchange databases	1) Studies published in peer-reviewed journals about burn injury care with an application that involved the transfer of visual images 2) No language restrictions	1) Studies not published in peer-reviewed journals or given as presentations2) Studies about technical comments relating to information technology for burn injury assessment without involving direct clinical care
Snoswell, C., Finnane, A., Janda, M., Soyer, H. P., & Whitty, J. A. (2016). Cost-effectiveness of store-and-forward teledermatology: a systematic review.* JAMA Dermatology*, *152*(*6*), 702-708.	No limit reported	1) CINAHL 2) Cochrane 3) EconLit 4) EMBASE 5) Google Scholar6) Medline 7) PubMed	1) Studies related to any population requiring dermatological care 2) Studies that include store-and-forward teledermatology intervention, regardless of the device or individual used to capture the images 3) Studies that compared the intervention with conventional face-to-face consultation 4) Studies which had outcomes expressed in terms of any kind of economic analysis 5) Only full-text journal articles available in English were included	None stated

**Fig. 2:**
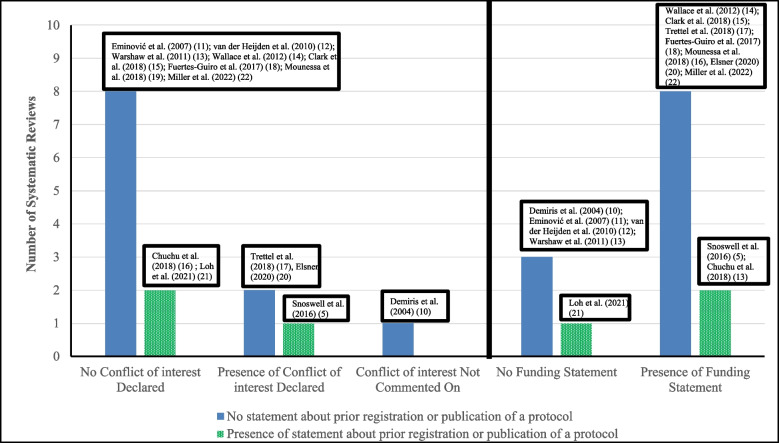
Protocol registration, Protocol publication, conflict of interest declaration, and funding statement reported in the Systematic Reviews

The number of electronic bibliographic databases used in the SRs ranged from 1 to 14 (Table 3). Five used hand-searching [9, 16, 19–21]. Only three SRs included a statement about prior registration or publication of a protocol [9, 22, 27]. All but one review [16] included a statement regarding any conflict of interest, and three reviews reported conflicts of interest [9, 23, 26]. Five reviews did not report a funding statement [16–19, 27], five reviews stated there was no financial support for the review [20, 23–25, 27, 28], and three reported receiving funding from medical councils or government agencies [9, 21, 22] (Fig. 2).

**Table 3:**
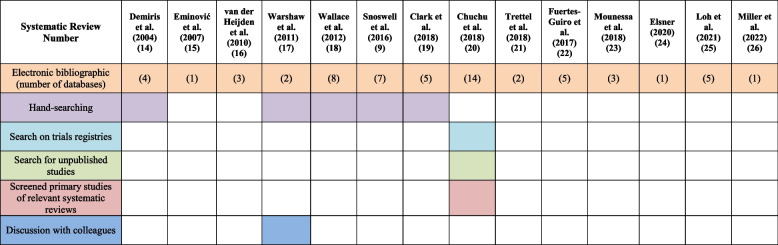
Information sources used in the 14 systematic reviews to identify primary studies

### Dermatological conditions

**Fig. 3:**
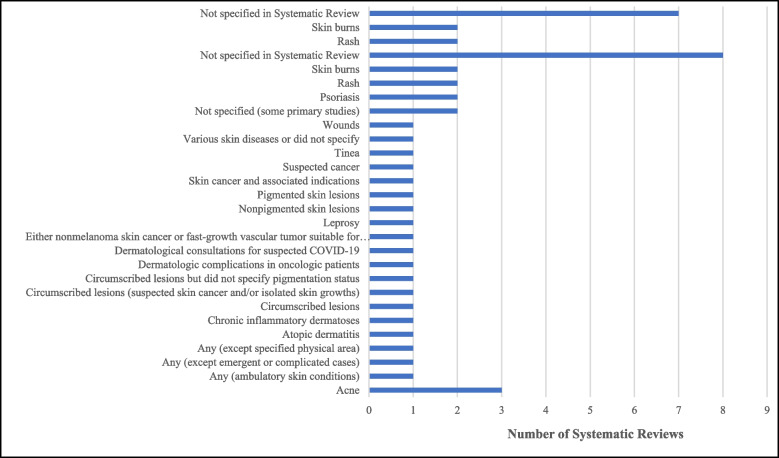
Dermatological conditions forming the focus of the systematic review

Eight of the SRs did not specify the dermatological conditions of interest [16–18, 21, 24, 25, 27, 28]. The remaining focused on burns [20, 22], rashes [19, 23], skin lesions [19], psoriasis [9, 23], skin cancer and other associated indications [22, 23], dermatologic complications amongst oncological patients [27], suspected malignant lesions [9], nonmelanoma skin cancer or fast-growth vascular tumor suitable for surgery under local anesthesia [9], acne [23], wounds [23], atopic dermatitis [23], tinea [23], leprosy [23], circumscribed lesions [19], pigmented and non-pigmented skin lesions [19], chronic inflammatory dermatoses [27], dermatological consultations for suspected COVID-19 [27], and any or unspecified conditions [9, 19, 23]. As a single diagnosis acne was addressed most often, it was featured specifically in four reviews (Fig. 3).

### Overlap of primary studies

The overall CCA was 4.9% which suggested a slight overlap of primary studies in the 14 included systematic reviews, and the overlap between pairs of studies ranged between 0.0% and 43.9%. Of the 91 pairs of systematic reviews, three pairs were categorized as having very high overlap (i.e., 15 and 17 = 43.9%; 17 and 21 = 25.9%; 15 and 21 = 22.2%) and four pairs were categorized as having high overlap (i.e., 24 and 25 = 13.3%; 17 and 20 = 12.4%; 14 and 15 = 10.7%; 19 and 21 = 10/1%). Nine pairs were categorized as having moderate overlap, and 75 pairs were categorized as having none to slight overlap (Table 4).

**Table 4:**
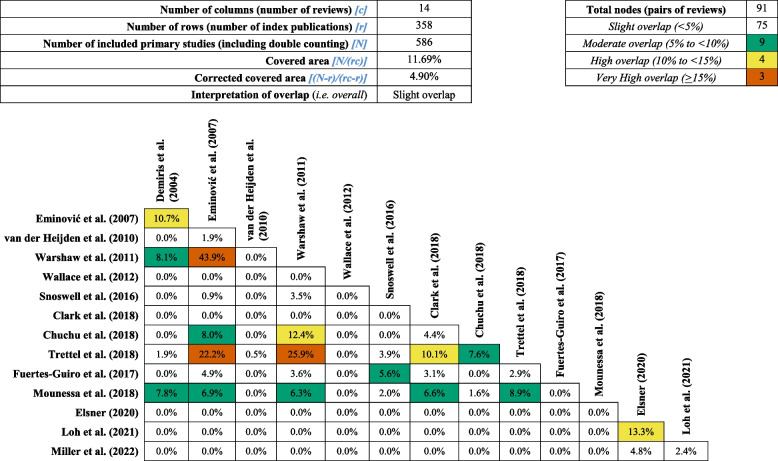
Analysis of overlap of primary studies in each of the 78 pairs of systematic reviews

### Quality assessment of the studies included in the systematic reviews

**Fig. 4:**
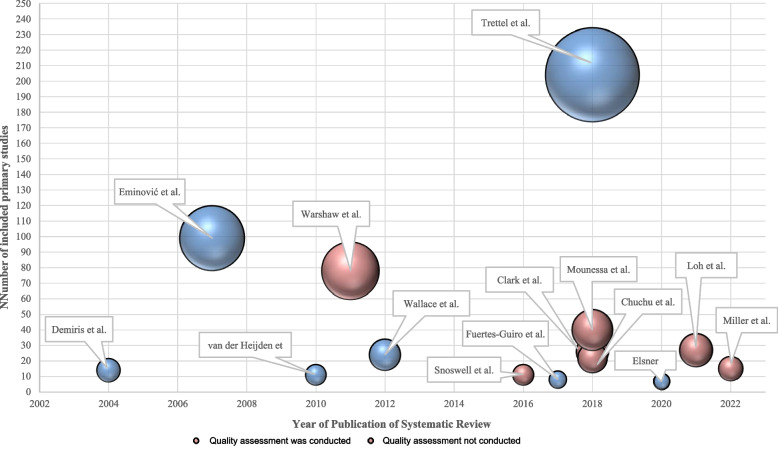
Overview of number of primary studies included and quality assessments conducted on primary studies

Seven of the 14 SRs reported conducting a quality assessment [9, 17, 19, 20, 23. 25, 26] (Fig. 4). Tools used were the Quality Assessment of Diagnostic Accuracy Studies (QUADAS and QUADAS-2) [19, 21, 22], the Consolidated Health Economic Evaluation Reporting Standards checklist (CHEERS) [9], the rating scheme provided by the Oxford Centre for Evidence-Based Medicine [28], and the abridged version [25]. In one instance the review [25] did not describe their findings in detail but commented on a low risk of bias in their included primary studies. Another review [27] did not report the quality assessment in the published article but did append their results on Mendeley Data (https://doi.org/10.17632/xd6ftfpgmc.1), while another did not report any quality assessment results [28].

Among the four reviews that reported quality assessment in detail, three described that at least half of the primary studies were at risk of bias [9, 19, 22]. The two reviews using the 14 QUADAS quality assessment made very different observations. In one, the proportion of primary studies that reported at least 10 QUADAS items was only 29% of 78 primary studies [19], in the other one it was 85% of 26 primary studies [21]. The systematic review that used the QUADAS-2 reported their findings in detail [22], highlighting that at least half of the 22 included primary studies were at high or unclear risk of bias for participant selection, reference standard, and flow and timing domains, while the majority were at low risk for the index test. In summary, they concluded that the quality of the studies included was of concern. Another systematic review of 11 primary studies [9] reported a wide range of quality scores using the CHEERS checklist (7 to 21 out of a total score of 24). The authors reported that the lower scores were due to a failure of the primary studies to report or discuss economic principles or justify the analytic approach used [9]. For the store and forward studies, the most relevant principles that were not included were study duration, appropriate financial conversions, and financial referencing.

### Main findings of two systematic reviews addressing cost/economic evaluation

Published in 2016 [9] and 2017 [24] these two reviews had a 5.6% overlap. Snoswell et al. [9] concluded that, while the evidence was sparse, SF teledermatology can be cost-effective when used as a triage mechanism to reduce the number of conventional face-to-face appointments. They identified three studies supporting the increasing cost-effectiveness of SF teledermatology when patients need to travel long distances to access dermatology services.

Fuertes-Guiro and Girrabent-Farres’s review [24] found that a teledermatology consultation requires more time (7.54 min extra) than a conventional consultation to make a diagnosis and management plan. In 2017 this represented an additional cost of 29.25 Euros for remote consultation; in addition, SF teledermatology was less costly than LI teledermatology. The authors observe that while there are some cost-utility and cost-effectiveness studies in the literature that indicate that telemedicine can reduce costs, these have not always been attentive to the cost of the dermatologist’s time, i.e., opportunity cost.

### Main findings of systematic reviews addressing the accuracy of telemedicine

One review [21] found that the diagnostic accuracy of mobile phone-based teledermatology was inferior to traditional face-to-face dermatology when comparing the clinical diagnosis with histopathology (weighted mean absolute difference 7.2%). Diagnostic concordance, defined as the agreement between teledermatology diagnosis and face-to-face teledermatology diagnosis, was generally good, and higher than the levels previously reported for SF. Only one study addressed management accuracy (matching management with histopathology) but found very high agreement when comparing the management decision based on teledermatology dermatoscopy and clinical images with histological-based management. Overall management concordance rates were very good, with a weighted average concordance of 80%. Whilst the review concluded that mobile teledermatology has yet to achieve a level of accuracy to replace conventional dermatology diagnosis, they described how over time mobile phone technology had developed for data capture, transmission, display, and storage improving the accessibility and convenience of mobile teledermatology.

The other systematic review (*n* = 22) addressed accuracy and focussed on teledermatology for detecting skin cancer in adults [22]. Data from four studies suggests that fewer than 7% of malignant skin lesions were missed by teledermatology. However, the applicability of these findings to the development of clinical services may be limited as participants were largely recruited from secondary or tertiary care clinics rather than the primary care setting where teledermatology is often used to triage and patients require referral to secondary care.

### Main findings of experiential systematic reviews of patient satisfaction

Demiris, Speedie, and Hicks looked at the quality of evidence about patient satisfaction with teledermatology [16]. They identified 13 primary studies that used self-administered questionnaires to measure patient satisfaction and one study that used phone interviews. The psychometric evaluation of the existing instruments was weak: content, construct, or reliability testing were not reported in any of the primary studies. Patients accepted teledermatology as a mode of care delivery but had concerns relating to privacy, embarrassment of being photographed, limited opportunities to express their problems and concerns, completeness of information transmitted, anxiety about the unfamiliar technology, and frustration with technical problems. The authors noted that the definition of satisfaction differed across the primary studies. They suggested that SF and LI need distinct evaluation tools.

Mounessa et al. reviewed 40 studies focussing on patient and provider satisfaction with SF and LI teledermatology [25]. Dissatisfaction with SF teledermatology was reported in 1 of 24 studies assessing patients and 3 out of 17 studies assessing teledermatology providers; it was noted that eight of these studies assessed both patient and teledermatology provider satisfaction with SF teledermatology. For SF services 96% of patients and 82% of providers were satisfied, and for LI teledermatology 89% of patients and 100% of providers were satisfied. It was noted that two LI teledermatology studies surveyed non-physician providers, and five studies included both patient and teledermatology providers.

### Main findings of combination-type review

One systematic review [19] was a combination of diagnostic test accuracy and cost/economic evaluation. Using 78 primary studies, Warshaw et al. [19] compared the diagnostic accuracy, clinical management, clinical outcomes, and the cost between teledermatology and clinic dermatology [19]. The authors reported that clinic dermatology had higher diagnostic accuracy than SF teledermatology (i.e., six studies, 19% better) and LI teledermatology (i.e., 11 studies, 11% better) that teledermatology accuracy rates improved by up to 15% with teledermatoscopy, and that the diagnostic concordance with clinic dermatology of SF teledermatology was good but better for LI teledermatology. Regarding management accuracy, the overall rates were similar but teledermatology and teledermatoscopy were inferior to clinic dermatology for malignant lesions. Regarding management concordance, rates were moderate to very good for both SF and LI teledermatology. The authors reported that there was insufficient evidence to evaluate the effect of teledermatology on clinical outcomes and that patient satisfaction and preferences for teledermatology were comparable with clinic dermatology. The time to treatment was significantly shorter and in-person visits to the dermatology clinic were avoided when patients had a teledermatology consultation. The SR reported that teledermatology was cost-effective compared to clinic dermatology on key considerations such as distance traveled by the patient, the volume of teledermatology, and the costs of clinic dermatology. However, the authors were unable to pool the data for analysis because these cost studies analyzed different outcome parameters.

### Main findings of scoping reviews

The first scoping review (*N* = 99 studies, 101 publications) aimed to describe the maturity status of teledermatology evaluation research and to explore the outcome measures [17]. It reported that while the number of feasibility studies increased, there was a lack of randomized controlled trials (RCTs), simulation cost studies, and post-implementation studies. Regarding outcome measures, the authors reported diagnostic accuracy as the most common (53 studies). Regarding study design, there were 43 intervention studies with the same patients as controls, 30 studies using an uncontrolled study design, 12 RCTs, seven intervention studies with different primary studies and patients as controls, and seven observational studies, SF teledermatology was most frequently used in the primary studies (62%), followed by LI teledermatology (30%), and combination of SF and LI teledermatology (2%). No data was available for the remaining studies.

The second scoping review (*N* = 11) aimed to provide an overview of the use of tertiary teledermatology [18], identifying four categories of tertiary teledermatology use: expertise (i.e., seeking advice from a dermatologist specialized in a specific area), continuing medical education (i.e., learning from other dermatologists), supervision of residents in training programs, and second opinion from dermatologists. The review identified three modalities of use (i.e. teledermatology consultation application in seven studies, website in two studies, and email list in one study). Regarding the type of teledermatology used, seven primary studies used SF teledermatology, and three used a combination of SF and LI teledermatology, but it was unclear what type of teledermatology was used in one study. Next, the authors reported that the outcome measure commonly reported was the effect of teledermatology on learning, followed by development cost, image quality, efficiency improvement, diagnostic validity, diagnostic reliability, diagnostic accuracy, patient satisfaction, and physician satisfaction.

The third scoping review included 24 primary studies and aimed to assess the evidence for the use of telemedicine in acute burn care and outpatient-based management [20]. Of the 24 included studies, seven studies evaluated clinical decision-making for acute burn care, eight studies assessed technical feasibility and clinical validation, and nine studies evaluated outpatient care. Wallace et al. [20] also reported that 14 primary studies assessed SF teledermatology, seven assessed LI teledermatology, and three assessed a combination of SF and LI teledermatology. This review found that teledermatology for burn care was rated as comparable to face-to-face assessment and as a tool that could improve clinical decision-making. The authors added that patients were satisfied and benefited from cost-savings in time and travel, but healthcare providers benefited from limited cost-savings only when a large volume of teledermatology was used. Regarding methodology, the authors commented that they did not find any RCTs, and of the 24 primary studies in their review, only 8 studies had controls. The primary studies in this review did not report a priori power calculation and were mainly subjective reports about teledermatology use rather than formal comparisons.

The fourth scoping review included in our evidence map review aimed to identify the use and current state of teledermatology across the world with regard to the geographical distribution of published studies, treated indications, research questions, and its reliability in diagnosis and therapy compared to classic face-to-face consultations [23]. Based on 204 primary studies included in this review, Trettel et al. [23] reported that the most common category of research questions posed by them was validity, concordance, or feasibility (*n* = 154), followed by effectiveness (i.e. comparison of teledermatology with face-to-face consultations; *n* = 33), costs, cost-effectiveness or cost–benefits of teledermatology (*n* = 24), quality of life (*n* = 4), and safety issues (*n* = 1). Regarding the comparison of teledermatology with face-to-face consultations, 138 studies reported that teledermatology was feasible, reliable, or effective under certain conditions, 34 studies found teledermatology to be superior to face-to-face consultations, 25 studies reported outcomes to be equivalent, and 15 studies reported outcomes to be inferior to face-to-face consultations. This scoping review included primary studies from a diverse range of clinical areas using teledermatology. Out of 204 primary studies, 127 studies reported either “various skin diseases” or did not specify them, 52 studies focused on skin cancer and associated diagnoses, 11 studies focused on wounds, 7 studies were on psoriasis, 4 studies were on atopic dermatitis, and single studies addressed acne, leprosy, rash, or tinea. Lastly, regarding the application of teledermatology, 105 primary studies were unspecified general evaluations, 59 studies were about patient management (e.g., referral from primary care physician to dermatologist) and triage, 23 studies were about the diagnosis or consultation of patients in remote locations, 17 studies were about the monitoring and consultation of patients in the nursing home or home care setting, and one study was about emergency diagnosis.

The fifth scoping review aimed to summarize teledermatology studies performed during the COVID-19 pandemic in 2020 [26]. Elsner [26] reported that two of the seven included studies were surveys among dermatologists showing that more than 80% offered teledermatology. The five remaining studies were retrospective cohort studies of low quality. Three of them investigated teledermatology in acne and inflammatory skin diseases, one the care of oncological patients with dermatological complications, and one teleconsultation in suspected COVID-19 cases. In all studies, teledermatology largely reduced the number of personal consultations. The review concludes that teledermatology could at least partly compensate for the limitations of in-person dermatological care during the COVID-19 pandemic.

The sixth scoping review included 27 primary studies and aimed to analyze the global utilization of teledermatology for patient care during the COVID-19 pandemic [27]. Out of 27 primary studies, 10 studies were about SF teledermatology, 6 studies were about LI teledermatology, 8 studies were about the combination of SF and LI teledermatology, and 3 studies did not specify the type of teledermatology used. Loh et al. [27] reported that teledermatology was useful in assessing and managing common ambulatory dermatoses. However, the authors highlighted concerns raised in the primary studies about low-quality images used in SF and LI teledermatology that reduced the accuracy of clinical assessments. During the COVID-19 pandemic, the authors reported that teledermatology decreased unnecessary face-to-face consultations, which reduced the risk of infections and the use of personal protective supplies. The authors also reported that teledermatology was used for the diagnosis of cutaneous manifestations of COVID-19 infection and the follow-up of onco-dermatology patients.

The final scoping review included 15 primary studies and aimed to identify the satisfaction levels of patients and providers of synchronous teledermatology during the COVID-19 pandemic, including the likelihood of patients and providers using teledermatology in the future [28]. Most studies reported that patients were willing to continue using synchronous teledermatology. Regarding satisfaction levels, Miller and Jones [28] reported that patients were satisfied with the patient–provider relationship and increased access to care. It was also noted that patients were generally satisfied with the technical quality and sound quality of their teledermatology consultation sessions. However, patients were reportedly not satisfied with the physical examination or quality compared with face-to-face care. As for the teledermatology providers, the authors reported that they were generally dissatisfied with the video or image quality and the quality of the teledermatology visit compared with face-to-face care. Despite these areas of dissatisfaction, it was noted that both the patients and providers were satisfied with visits meeting patient needs. The authors also observed that most questions asked when assessing satisfaction levels focused on quality of care and technical aspects of teledermatology, rather than access to care, overall satisfaction, and the patient-provider relationship.

## Discussion

### Main findings of the evidence map of teledermatology

Our evidence map review identified 14 systematic reviews published between 2004 and 2022, that were from Western countries with the exception of one from Singapore. LI teledermatology is more costly than SF teledermatology. SF teledermatology is cost-effective as a triage mechanism to reduce face-to-face consultations but dermatologists reportedly spend more time during teledermatology consultations than in-person consultations [9, 24]. Mobile teledermatology has good diagnostic concordance with face-to-face dermatology when used in a tertiary setting; there remains a lack of data to support its use for triage in the primary care setting [22]. Although the accessibility and convenience of mobile teledermatology have improved, there is a lack of evidence to support it replacing face-to-face dermatology [21, 22]. Most patients and service providers were satisfied with SF and LI teledermatology [25] but have concerns about privacy, communication (accuracy and completeness) with the doctor, and technical requirements to use the service [16]. The accuracy of teledermatology increases with teledermatoscopy, but face-to-face dermatology had higher diagnostic and management accuracy than SF and LI teledermatology [19]. LI teledermatology was also reported to have higher diagnostic concordance than SF teledermatology, while management concordance was rated as moderate to very good for LI and SF teledermatology. Teledermatology was also reported to be cost-effective compared to face-to-face dermatology when considering the distance traveled by the patient, volume of teledermatology consultations, and costs of operating clinic dermatology.

Clinical areas where teledermatology was commonly researched were skin cancer, wounds, psoriasis, atopic dermatitis, acne, leprosy, rash, and tinea [17, 18, 20, 23, 26, 27]. The application of teledermatology included general evaluations, patient management and triage, diagnosis, consultation, or monitoring in remote locations, nursing homes, or home care settings [17, 18, 20, 23, 26, 27]. During the COVID-19 pandemic, most healthcare professionals reported using teledermatology as an alternative to face-to-face consultations, to minimize the risk of infections and reduce the need to use personal protective supplies [17, 18, 20, 23, 26–28].

### Gaps in the literature highlighted

While feasibility studies are common, there is a lack of RCTs, simulation cost studies, and post-implementation studies, all methodologies that researchers could consider when designing future studies. There was a high level of heterogeneity in the study methodology, the skin conditions included, and the outcome parameters used. Systematic reviews were unable to pool the data for analysis and draw generalizable conclusions. Although there was a large number of studies that assessed patient and provider satisfaction with teledermatology, the definition of satisfaction differed between studies. There is a paucity of studies that address in detail the reasons for dissatisfaction, and yet this is a fundamental requirement when developing interventions to improve satisfaction. Most studies compared the diagnostic accuracy, diagnostic concordance, management accuracy, and management concordance of teledermatology to care by a specialist dermatologist, there is a lack of studies that compare teledermatology with dermatologic care provided by non-dermatologists (e.g., primary care). There were no systematic reviews of articles addressing the safety of teledermatology; safety includes the clinical aspect of teledermatology, but also the security of data exchanged during teledermatology, especially since this is a concern that has been highlighted by patients in different studies. Next, the systematic reviews included in our evidence map all originated from developed countries. This uneven global distribution of manuscript origin is not dissimilar to the findings of a bibliometric analysis of teledermatology publications between 1980 and 2013, which found the top three countries were the USA, the UK, and Australia [30]. Teledermatology may be particularly beneficial in countries where the distances between health care facilities are large, where transport is difficult, and where specialist care is scarce. Next, there seems to be a gap in the literature regarding the effects on work processes and workflows due to the implementation of teledermatology in a clinic. While teledermatology is meant to help healthcare professionals, there might be greater indirect costs and opportunity costs that may deem teledermatology to be less cost-effective.

### Strengths and limitations of our overview

The broad search strategy and stringent screening processes used give confidence that the map of teledermatology evidence created reflects the current state of the teledermatology literature. We searched registries to look for any unpublished studies. However, a limitation is the exclusion of reviews published in languages other than English. Another limitation is that the inter-rater agreement could have been recorded.

### Recommendations and implications

The heterogeneity of outcomes addressed, and the outcome measurement instruments used limit the pooling of data. Moving forward it would be beneficial to develop a core outcome set for teledermatology research [31]. Secondly, as technology advances, research about the accuracy, concordance, cost, and safety of teledermatology needs to be updated, to confirm that the technological advances bring clinical benefit and are cost-effective. There is a lack of studies that compare teledermatology with dermatologic care provided by non-dermatologists (e.g., primary care). Fourth, future teledermatology studies should include non-Western low and middle-income countries, to assess the utility and feasibility of teledermatology in areas that may require it most (e.g., remote areas where patients have to travel long distances for dermatological care). Lastly, future studies could include patient involvement as part of the study design as this may lead to better-designed research that is more relevant with clearer outcomes.

## Conclusions

Teledermatology, leveraging technology for remote dermatological consultations, aims to enhance access, reduce costs, and improve health outcomes. This evidence map reviews 14 systematic reviews (2004–2022) to understand teledermatology’s landscape. Advantages include overcoming barriers to care and cost-effectiveness, particularly in triaging face-to-face appointments. However, the evidence is heterogeneous, lacking robust research across diverse conditions, settings, and patient groups. Asynchronous (store and forward) and real-time consultations prevail. Teledermatology’s benefits encompass shorter waiting times, cost-effectiveness, and comparable diagnostic concordance with face-to-face consultations. The review identifies gaps, emphasizing the need for more randomized controlled trials, standardized outcome measures, and exploration of non-Western contexts. While patient and provider satisfaction is generally positive, concerns persist about privacy, communication, and technical aspects. Notably, teledermatology’s role during the COVID-19 pandemic is acknowledged, reducing in-person visits and preserving resources. The review suggests future research should address dissatisfaction reasons, safety concerns, and global disparities in teledermatology literature, urging inclusivity and patient involvement for comprehensive insights.

## Supplementary Information

Supplementary Material 1: Appendix S1. Search strategy.

## Data Availability

The datasets used and analyzed during the current study are available from the corresponding author on reasonable request.

## Abbreviations

CCA: Corrected covered area
GROOVE: Graphical Representation of Overlap for OVErviews
LI: Live-interactive teledermatology
RCT: Randomized controlled trial
SF: Store and forward teledermatology
