# Supplementary material for: Teledermatology: an evidence map of systematic reviews
Source: Syst Rev. 2024 Oct 12;13:258. doi: 10.1186/s13643-024-02655-5 (PMC11476646; doi:10.1186/s13643-024-02655-5)
Supplement: Supplementary file 1 — Supplementary Material 1: Appendix S1. Search strategy. [file 13643_2024_2655_MOESM1_ESM.docx]

# SUPPLEMENTAL APPENDICES

## APPENDIX S1. Search strategy

| **Database:** | EBSCO CINAHL |
| --- | --- |
| **Search Strategy:** | 1. AB teledermatology OR TI teledermatology OR TX teledermatology 2. AB dermatology OR TI dermatology OR TX dermatology 3. AB (telemedicine OR telehealth OR telecare) OR TI (telemedicine OR telehealth OR telecare) OR TX (telemedicine OR telehealth OR telecare) 4. (S2 AND S3) 5. AB telederm* OR TI telederm* OR TX telederm* 6. (S1 OR S4 OR S5) 7. AB asynchronous OR TI asynchronous OR TX asynchronous 8. AB synchronous OR TI synchronous OR TX synchronous 9. AB (store-and-forward OR "store-and-forward" OR store and forward OR "store and forward") OR TI (store-and-forward OR "store-and-forward" OR store and forward OR "store and forward") OR TX (store-and-forward OR "store-and-forward" OR store and forward OR "store and forward") 10. AB ("real time" OR real time OR real-time OR "real-time") OR TI ("real time" OR real time OR real-time OR "real-time") OR TX ("real time" OR real time OR real-time OR "real-time") 11. (S7 OR S8 OR S9 OR S10) 12. (S6 AND S11) 13. AB (Systematic review*) OR TI (Systematic review*) OR TX (Systematic review*) 14. AB (Meta-analysis OR Meta analysis) OR TI (Meta-analysis OR Meta analysis) OR TX (Meta-analysis OR Meta analysis) 15. (S13 OR S14) 16. (S12 AND S15) |
| **Database:** | Ovid EMBASE |
| **Search Strategy:** | 1. teledermatology.ab,ti,tw. 2. dermatology.ab,ti,tw. 3. (telemedicine or telehealth or telecare).ab,ti,tw. 4. 2 AND 3 5. telederm*.ab,ti,tw. 6. 1 OR 4 OR 5 7. asynchronous.ab,ti,tw. 8. synchronous.ab,ti,tw. 9. (store-and-forward or "store-and-forward" or (store and forward) or "store and forward").ab,ti,tw. 10. ("real time" or real time or real-time or "real-time").ab,ti,tw. 11. 7 OR 8 OR 9 OR 10 12. 6 AND 11 13. Systematic review*.ab,ti,tw. 14. (Meta-analysis OR Meta analysis).ab,ti,tw. 15. 13 OR 14 16. 12 AND 15 |
| **Database:** | PubMed |
| **Search Strategy:** | 1. (teledermatology[Title/Abstract]) OR teledermatology[Text Word] 2. (dermatology[Title/Abstract]) OR dermatology[Text Word] 3. ((telemedicine[Title/Abstract] OR telehealth[Title/Abstract] OR telecare[Title/Abstract])) OR (telemedicine[Text Word] OR telehealth[Text Word] OR telecare[Text Word]) 4. (((dermatology[Title/Abstract]) OR dermatology[Text Word])) AND (((telemedicine[Title/Abstract] OR telehealth[Title/Abstract] OR telecare[Title/Abstract])) OR (telemedicine[Text Word] OR telehealth[Text Word] OR telecare[Text Word])) 5. (telederm*[Title/Abstract]) OR telederm*[Text Word] 6. ((((teledermatology[Title/Abstract]) OR teledermatology[Text Word])) OR ((((dermatology[Title/Abstract]) OR dermatology[Text Word])) AND (((telemedicine[Title/Abstract] OR telehealth[Title/Abstract] OR telecare[Title/Abstract])) OR (telemedicine[Text Word] OR telehealth[Text Word] OR telecare[Text Word])))) OR ((telederm*[Title/Abstract]) OR telederm*[Text Word]) 7. (asynchronous[Title/Abstract]) OR asynchronous[Text Word] 8. (synchronous[Title/Abstract]) OR synchronous[Text Word] 9. (((store-and-forward[Title/Abstract] OR "store-and-forward"[Title/Abstract] OR (store[Title/Abstract] AND forward)[Title/Abstract] OR "store[Title/Abstract] AND forward")[Title/Abstract])) OR ((store-and-forward[Text Word] OR "store-and-forward"[Text Word] OR (store[Text Word] AND forward)[Text Word] OR "store[Text Word] AND forward")[Text Word]) 10. ((("real time"[Title/Abstract] OR real time[Title/Abstract] OR real-time[Title/Abstract] OR "real-time")[Title/Abstract])) OR (("real time"[Text Word] OR real time[Text Word] OR real-time[Text Word] OR "real-time")[Text Word]) 11. (((((asynchronous[Title/Abstract]) OR asynchronous[Text Word])) OR ((synchronous[Title/Abstract]) OR synchronous[Text Word])) OR ((((store-and-forward[Title/Abstract] OR "store-and-forward"[Title/Abstract] OR (store[Title/Abstract] AND forward)[Title/Abstract] OR "store[Title/Abstract] AND forward")[Title/Abstract])) OR ((store-and-forward[Text Word] OR "store-and-forward"[Text Word] OR (store[Text Word] AND forward)[Text Word] OR "store[Text Word] AND forward")[Text Word]))) OR (((("real time"[Title/Abstract] OR real time[Title/Abstract] OR real-time[Title/Abstract] OR "real-time")[Title/Abstract])) OR (("real time"[Text Word] OR real time[Text Word] OR real-time[Text Word] OR "real-time")[Text Word])) 12. ((((((teledermatology[Title/Abstract]) OR teledermatology[Text Word])) OR ((((dermatology[Title/Abstract]) OR dermatology[Text Word])) AND (((telemedicine[Title/Abstract] OR telehealth[Title/Abstract] OR telecare[Title/Abstract])) OR (telemedicine[Text Word] OR telehealth[Text Word] OR telecare[Text Word])))) OR ((telederm*[Title/Abstract]) OR telederm*[Text Word]))) AND ((((((asynchronous[Title/Abstract]) OR asynchronous[Text Word])) OR ((synchronous[Title/Abstract]) OR synchronous[Text Word])) OR ((((store-and-forward[Title/Abstract] OR "store-and-forward"[Title/Abstract] OR (store[Title/Abstract] AND forward)[Title/Abstract] OR "store[Title/Abstract] AND forward")[Title/Abstract])) OR ((store-and-forward[Text Word] OR "store-and-forward"[Text Word] OR (store[Text Word] AND forward)[Text Word] OR "store[Text Word] AND forward")[Text Word]))) OR (((("real time"[Title/Abstract] OR real time[Title/Abstract] OR real-time[Title/Abstract] OR "real-time")[Title/Abstract])) OR (("real time"[Text Word] OR real time[Text Word] OR real-time[Text Word] OR "real-time")[Text Word]))) 13. (Systematic review*[Title/Abstract]) OR Systematic review*[Text Word] 14. (((Meta-analysis[Title/Abstract] OR Meta analysis)[Title/Abstract])) OR ((Meta-analysis[Text Word] OR Meta analysis)[Text Word]) 15. (((Systematic review*[Title/Abstract]) OR Systematic review*[Text Word])) OR ((((Meta-analysis[Title/Abstract] OR Meta analysis)[Title/Abstract])) OR ((Meta-analysis[Text Word] OR Meta analysis)[Text Word])) 16. ((((((((teledermatology[Title/Abstract]) OR teledermatology[Text Word])) OR ((((dermatology[Title/Abstract]) OR dermatology[Text Word])) AND (((telemedicine[Title/Abstract] OR telehealth[Title/Abstract] OR telecare[Title/Abstract])) OR (telemedicine[Text Word] OR telehealth[Text Word] OR telecare[Text Word])))) OR ((telederm*[Title/Abstract]) OR telederm*[Text Word]))) AND ((((((asynchronous[Title/Abstract]) OR asynchronous[Text Word])) OR ((synchronous[Title/Abstract]) OR synchronous[Text Word])) OR ((((store-and-forward[Title/Abstract] OR "store-and-forward"[Title/Abstract] OR (store[Title/Abstract] AND forward)[Title/Abstract] OR "store[Title/Abstract] AND forward")[Title/Abstract])) OR ((store-and-forward[Text Word] OR "store-and-forward"[Text Word] OR (store[Text Word] AND forward)[Text Word] OR "store[Text Word] AND forward")[Text Word]))) OR (((("real time"[Title/Abstract] OR real time[Title/Abstract] OR real-time[Title/Abstract] OR "real-time")[Title/Abstract])) OR (("real time"[Text Word] OR real time[Text Word] OR real-time[Text Word] OR "real-time")[Text Word]))))) AND ((((Systematic review*[Title/Abstract]) OR Systematic review*[Text Word])) OR ((((Meta-analysis[Title/Abstract] OR Meta analysis)[Title/Abstract])) OR ((Meta-analysis[Text Word] OR Meta analysis)[Text Word]))) |
| **Database:** | Elsevier Scopus |
| **Search Strategy:** | 1. TITLE-ABS-KEY (teledermatology) 2. TITLE-ABS-KEY (dermatology) 3. TITLE-ABS-KEY (telemedicine OR telehealth OR telecare) 4. #2 AND #3 5. TITLE-ABS-KEY (telederm*) 6. #1 OR #4 OR #5 7. TITLE-ABS-KEY (asynchronous) 8. TITLE-ABS-KEY (synchronous) 9. TITLE-ABS-KEY ((store-and-forward) OR ("store-and-forward") OR (store and forward) OR ("store and forward")) 10. TITLE-ABS-KEY (("real time") OR (real time) OR (real-time) or ("real-time")) 11. #7 OR #8 OR #9 OR #10 12. #6 AND #11 13. TITLE-ABS-KEY (Systematic review*) 14. TITLE-ABS-KEY (Meta-analysis OR Meta analysis) 15. #13 OR #14 16. #12 AND #15 |
| **Database:** | Web of Science |
| **Search Strategy:** | 1. ALL=(teledermatology) 2. ALL=(dermatology) 3. ALL=(telemedicine OR telehealth OR telecare) 4. #2 AND #3 5. ALL = (telederm*) 6. #1 OR #4 OR #5 7. ALL = (asynchronous) 8. ALL = (synchronous) 9. ALL = ((store-and-forward) OR ("store-and-forward") OR (store and forward) OR ("store and forward")) 10. ALL = (("real time") OR (real time) OR (real-time) OR ("real-time")) 11. #7 OR #8 OR #9 OR #10 12. #6 AND #1 13. ALL = (Systematic review*) 14. ALL = ((Meta-analysis) OR (Meta analysis)) 15. #13 OR #14 16. #12 AND #15 |
| **Database:** | Cochrane Library |
| **Search Strategy:** | 1. (teledermatology).ab,ti,tw. 2. (dermatology).ab,ti,tw. 3. (((telemedicine) OR (telehealth) OR (telecare))).ab,ti,tw. 4. #2 AND #3 5. (telederm*).ab,ti,tw. 6. #1 OR #4 OR #5 7. asynchronous.ab,ti,tw. 8. synchronous.ab,ti,tw. 9. (store-and-forward or "store-and-forward" or (store and forward) or "store and forward").ab,ti,tw. 10. ("real time" or real time or real-time or "real-time").ab,ti,tw. 11. #7 OR #8 OR #9 OR #10 12. #6 AND #11 13. Systematic review*.ab,ti,tw. 14. (Meta-analysis OR Meta analysis).ab,ti,tw. 15. #13 OR #14 16. #12 AND #15 |
| **Database:** | JBI Database of Systematic Reviews and Implementation Reports |
| **Search Strategy:** | 1. teledermatology |
| **Database:** | OpenGray |
| **Search Strategy:** | 1. teledermatology |
